# Burden attributable to Cardiometabolic Diseases in Zimbabwe: a retrospective cross-sectional study of national mortality data

**DOI:** 10.1186/s12889-015-2554-z

**Published:** 2015-12-07

**Authors:** Mutsa P. Mutowo, Alice J. Owen, Baki Billah, Paula K. Lorgelly, Kudzai E. Gumbie, John C. Mangwiro, Andre M. N. Renzaho

**Affiliations:** School of Public Health and Preventive Medicine, Monash University, Melbourne, 3004 Australia; Centre for Health Economics, Monash University, Melbourne, 3004 Australia; Fellow of the Institute of Actuaries (FIA) , Harare, Zimbabwe; Zimbabwe Diabetes Association, PO Box 1797, Harare, Zimbabwe; School of Social Science and Psychology, University of Western Sydney, Sydney, 2751, New South Wales Australia

**Keywords:** Cardiometabolic diseases, Non-communicable diseases, Zimbabwe

## Abstract

**Background:**

Cardiometabolic diseases (CMDs) are an important cause of mortality worldwide and the burden associated with them is increasing in Sub-Saharan Africa. The tracking of mortality helps support evidence based health policy and priority setting. Given the growing prevalence of non-communicable diseases in Zimbabwe, a study was designed to determine the mortality attributable to CMDs in Zimbabwe.

**Methods:**

The study design was a retrospective cross-sectional analysis of national mortality from 1996 to 2007, collated by the Ministry of Health and Child Welfare in Zimbabwe. We employed generalized additive models to flexibly estimate the trend of the CMD mortality and a logistic regression model was used to find significant factors (cause of death according to the death certificate) of the CMD mortality and predict CMD mortality to 2040.

**Results:**

CMDs accounted for 8.13 % (95 % CI: 8.08 % - 8.18 %) of all deaths during 1996 to 2007 (*p* = 0.005). During the study period CMD mortality rate increased by 29.4 % (95 % CI: 19.9 % - 41.1 %). The association between gender and CMD mortality indicated female mortality was higher for diabetes (*p* < 0.001), hypertensive disease (*p* < 0.001), CVD (*p* < 0.001) and pulmonary disease (*p* < 0.001), while male mortality was higher for ischaemic (*p* = 0.010) and urinary diseases (*p* < 0.001). There was no gender difference for endocrine disease (*p* = 0.893). Overall, females have 1.65 % higher mortality than males (*p* < 0.001). CMD mortality is predicted to increase from 9.6 % (95 % CI: 8.0 % - 11.1 %) in 2015 to 13.7 % (95 % CI: 10.2 % - 17.2 %) in 2040 for males, and from 11.6 % (95 % CI: 10.2 % - 12.9 %) in 2015 to 16.2 % (95 % CI: 13.1 % - 19.3 %) in 2040 in females.

**Conclusion:**

The findings of this study indicate a growing prevalence of CMDs and related mortality in Zimbabwe. Health policy decisions and cost-effective preventive strategies to reduce the burden of CMDs are urgently required.

## Background

Cardiometabolic disease (CMD) is an emerging term that encompasses cardiovascular disease, diabetes and metabolic syndrome (MetS). The primary components of MetS include obesity, insulin resistance, dyslipidemia, and hypertension [1–4], which are major risk factors for diabetes, and vascular diseases including myocardial infarction, stroke, chronic kidney disease, and ischemic gangrene [1–4]. CMDs are becoming increasingly common in Sub-Saharan African (SSA) countries [5–7], with the prevalence ranging from non-existent to as high as about 50 % or even higher depending on the population setting [8, 9]. The prevalence of CMDs in SSA is thought to be driven by the departure from traditional African lifestyles and diets in favour of Western lifestyles and diets [8]. Changes to lifestyle including rapid urbanization, reductions in occupational physical activity, increased intake of highly processed foods and abdominal obesity are considered risk factors for CMD [8]. Mortality attributable to CMDs could therefore be targeted through public health interventions.

The pattern of morbidity and mortality in Zimbabwe, like most of SSA, has been dominated by infectious and parasitic diseases, nutritional deficiencies, obstetric causes and perinatal conditions, as well as injury-related conditions [9]. However, CMDs are placing an increasing burden on health care systems in many countries in SSA including Zimbabwe, with CMD mortality ranging from 17 % in Zimbabwe, Zambia and Mozambique to 34 % in South Africa and 36 % in Cameroon [9]. Hypertension was ranked first and diabetes ranked fourth (after asthma and epilepsy) amongst the non-communicable disease (NCD) outpatient visits recorded in Zimbabwean public hospitals [10], and while infections are still a major health burden in Zimbabwe, NCDs have also become a problem.

In Zimbabwe, cause of death statistics are the primary data source for monitoring the health status and informing chronic disease priorities in the country [10]. However NCD mortality estimates for Zimbabwe have a high degree of uncertainty because they are not based on any systematically collected national NCD mortality data. The estimates are based on a combination of African country life tables, cause of death models, regional cause of death patterns, and program estimates for some major causes of death (not including NCDs) [11]. A Zimbabwean burden of disease study [12] illustrated the importance of conducting this kind of analysis for a specific country, rather than assuming that regional estimates would provide plausible estimates, when it reported that disease patterns in Zimbabwe differed substantially from regional estimates. A meta-analysis study found the pooled prevalence of diabetes to be 5.7 % and hypertension to be 30 % in Zimbabwe [13, 14]. The purpose of this paper was to estimate the mortality attributable to CMDs in Zimbabwe, using the death certificate forms registered through the Zimbabwe birth and deaths registry and assembled by the Zimbabwe National Statistics Agency (Zimstat) for the total deaths in the country covering the period 1996 to 2007.

## Methods

Gender-specific mortality and population data from Zimbabwe National Statistics Agency (Zimstat) and the Ministry of Health and Child Care (MoHCC) in Zimbabwe for the period 1996 to 2007 were available, although data for 1998 were missing. Data were stored on an electronic system in the Zimstat offices in Harare, and downloaded after approval was granted by the MoHCC. Gender-specific death rates for CMDs were based on deaths with a mention of diabetes mellitus, other endocrine & metabolic diseases, hypertensive disease, ischaemic heart disease, cardio and cerebrovascular accidents, pulmonary circulation, other circulatory and heart disease, and urinary system diseases (which includes chronic kidney disease) as the underlying cause of death from the total deaths recorded from 1996 to 2007. The longitudinal nature of the data also allowed us to investigate trends in CMD mortality.

### Classification of the causes of death

Zimbabwe used the ninth revision of the International Classification of Disease (ICD-9) coding from 1996 to 2003, and tenth revision ICD (ICD-10) coding from 2004 to 2007 [15, 16]. The codes collected for the causes of death are listed in Table 1. Data using ICD-9 codes were converted to ICD-10 codes with the ICD-10 Translator [17].

**Table 1:** ICD-9 and ICD-10 codes for cause of death listed on death certificates

Cause of death	ICD-9	ICD-10
Diabetes	249-251.2	E10-E14
Hypertensive diseases	401-405	I10-I15
Ishaemic Heart Disease	410-414	I20-I25
Cardio- and Cerebrovascular Disease	430-438	I60-I69
Pulmonary Heart Disease and other diseases of the heart and circulatory system	415-417, 420-429, 440-448, 451-459	I26-I28, I30-I52, I70-I99
Urinary systems Diseases	580-599	N00-N39
Endocrine and Metabolic Diseases	270-279	E15-E16, E70-E90

### Statistical analysis

A Generalized Additive Model (GAM) was used to analyse the trend, as the relationship between mortality and time was not linear. This model is suitable for exploring the data and visualizing the relationship between the outcome variable and the covariates [18]. A smoothing function was used for summarizing the trend of the outcome as a function of time adjusted for gender, where the smoothing function does not assume a rigid form for the dependence of outcome. A p-value of less than 5 % was considered as statistically significant.

A logistic regression was used to predict CMD mortality for the male and female population from 2015 to 2040. The 25-year selected period is the most common length of select period used for mortality projections [19], and will assist in quantifying reductions in mortality for the UN sustainable development health goals for 2030 [20]. We assumed that there would be no collinearity between gender and time. The projections do not take explicit account of changing population demographics during the study period, age, geographic locations, standards of living, socio-economic conditions of the population and access to health care services. Additionally, trends in major risk factors such as tobacco smoking, alcohol consumption and overweight and obesity have not been accounted for.

## Results

The population mortality from 1996 to 2007 is shown in Table 2. A total of 942,319 deaths were registered from 1996 to 2007 (note that no records are available for 1998). Approximately one in ten deaths (8.13 % 95 % CI: 8.08 % - 8.18 %) was attributable to CMD. Over the 12 year study period, CMD mortality rate increased by 29.4 % (95 % CI: 19.9 % - 41.1 %).

**Table 2:** Number of deaths for each year of death according to the population death register: 1996-2007 (missing 1998)

Year	CMD Deaths	Total deaths	Total Popn	Crude mortality rate		% Death accounted for by CMD	
				(/100,000)	95 % CI	%	95 % CI
1996	6088	89207	11,846,110	753.0	748.1 - 758.0	6.8	6.7 - 7.0
1997	6422	85964	12,045,813	713.6	708.9 - 718.4	7.5	7.3 - 7.7
1999	7290	91517	12,384,727	739.0	734.2 - 743.7	8.0	7.8 - 8.2
2000	7352	96163	12,503,652	769.1	764.2 - 773.9	7.6	7.5 - 7.8
2001	7468	98946	12,586,763	786.1	781.2 - 791.0	7.5	7.4 - 7.7
2002	8098	99820	12,640,922	789.7	784.8 - 794.6	8.1	7.9 - 8.3
2003	6827	88946	12,673,103	701.8	697.2 - 706.5	7.7	7.5 - 7.9
2004	8929	103848	12,693,047	818.1	813.2 - 823.1	8.6	8.4 - 8.8
2005	8553	92516	12,710,589	727.9	723.2 - 732.6	9.2	9.0 - 9.4
2006	8958	95717	12,724,308	752.2	747.5 - 757.0	9.4	9.2 - 9.6
2007	8149	92185	12,740,160	723.6	718.9 - 728.2	8.8	8.7 - 9.0
Total Period (excl. 1998)	84134	1034829				8.13	8.08 - 8.18

**Fig. 1:**
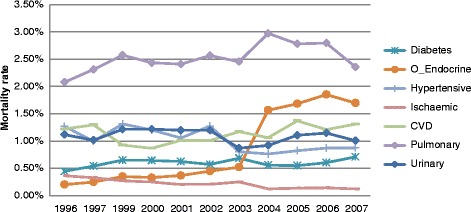
The proportion of deaths in males due to CMDs categories, 1996-2007

**Fig. 2:**
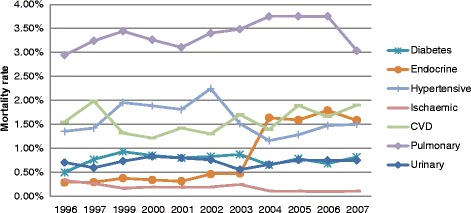
The proportion of deaths in females due to CMDs categories, 1996-2007

**Fig. 3:**
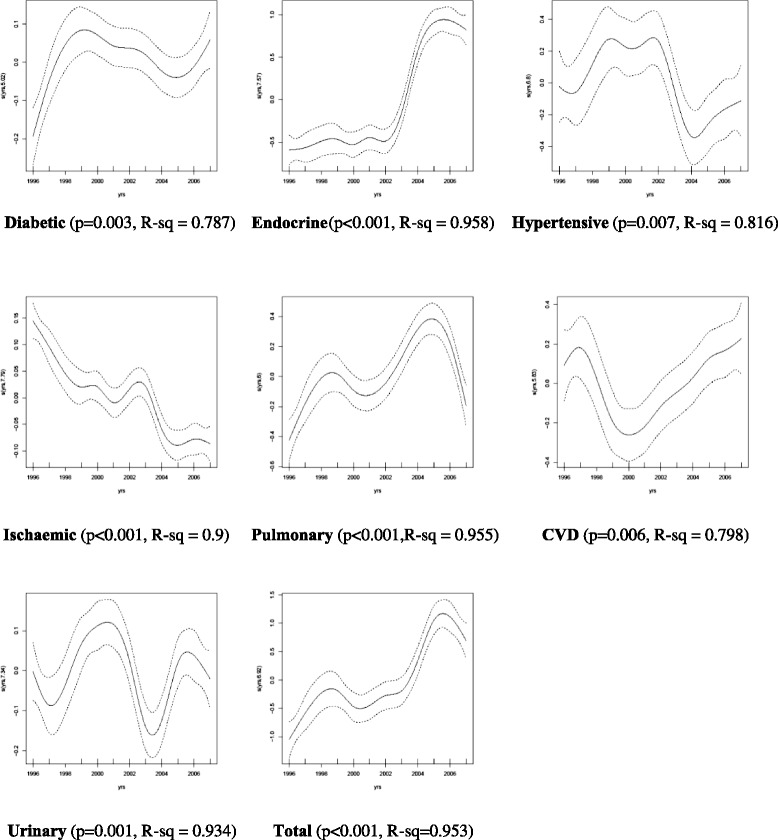
Estimate of smooth function of time adjusted for gender, evaluated at the observed time using the Generalized Additive Model

The mortality rates of the seven causes of death comprising CMDs from 1997 to 2007 appeared to follow similar patterns in males and females (Figs. 1 and 2). Pulmonary heart disease and other diseases of the heart and circulatory system, CVD, diabetes and hypertensive diseases made the largest contribution to CMD mortality, with females having a higher mortality rate than males. The results of the GAM analysis (Fig. 3) show the trends of mortality for diabetes mellitus, other endocrine & metabolic diseases, hypertensive disease, ischaemic heart disease, cardio and cerebrovascular accidents, pulmonary circulation, other circulatory and heart disease, urinary system diseases, as well as the total CMD mortality. The solid line in the middle represents the smoothing trend line, while the dashed lines represent the 95 % confidence interval of the trend.

The clear contrast in these figures is the substantial cause-specific differences over the study period. For example, diabetes mortality rose sharply until 1997, then again from 2005, whereas endocrine mortality was low from 1996 and rose sharply from 2002. Cardiovascular deaths decreased steadily from 1996, but rise sharply from 2000. Finally, total CMD mortality shows a clear increase peaking at 2006. The *p*-value for each cause of death is also presented in Fig. 3 and shows a strong significance with the smoothing trend, while the corresponding adjusted R^2^ value shows a very high percentage of variation in the outcome, explained by time and gender, demonstrating a strong relationship between mortality and the covariates. The GAM analysis indicated that females had higher mortality rates for diabetes (*p* < 0.001), hypertensive disease (*p* < 0.001), CVD (*p* < 0.001) and pulmonary disease (*p* < 0.001) during the period. However, males had a higher mortality for ischaemic (*p* < 0.00966) and urinary diseases (*p* < 0.001). There was no gender difference for endocrine diseases (*p* = 0.893).

**Fig. 4:**
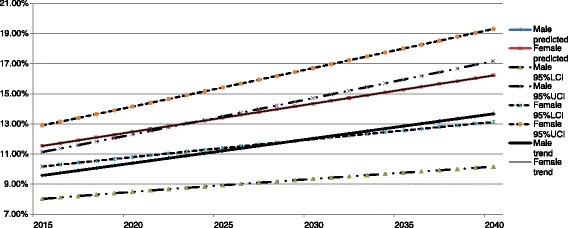
Projected CMD mortality for males and females in Zimbabwe for the period 2015 to 2040

CMD mortality is predicted to increase from 9.6 % (95 % CI: 8.0 % - 11.1 %) in 2015 to 13.7 % (95 % CI: 10.2 % - 17.2 %) in 2040 for males, and from 11.6 % (95 % CI: 10.2 % - 12.9 %) in 2015 to 16.2 % (95 % CI: 13.1 % - 19.3 %) in 2040 in females (see Fig. 4).

## Discussion

The pattern of CMD mortality increasing over the study period from 1996 to 2007 is consistent with observations that CMD prevalence is reaching epidemic proportions in Africa [21]. Diseases of the urinary system, which include renal/kidney disease, are often associated with cardiovascular risk factors, including dyslipidemia, hypertension, and smoking [22]. Zimbabwean males are nine times more likely to consume tobacco products than females, which could explain the higher mortality of urinary system diseases (including kidney disease) in Zimbabwean males [23]. There was no gender difference found in mortality attributable to endocrine diseases, but there was a significant increase in mortality from 2004. In 2004 the Zimbabwean government initiated its national Antiretroviral therapy (ART) programme for HIV/AIDS (Human Immunodeficiency Virus/ Acquired Immune Deficiency Syndrome) [24], however due to the political and economic instability at the time, mass emigration of health workers and inadequate availability of foreign exchange reserves meant procurement and distribution systems for drugs and supplies was fragmented [25]. The use of ART has been associated with adverse endocrine dysfunction [26], with adrenal insufficiency the most common HIV endocrinopathy found present in 77 % of post-mortems [27]. As Zimbabwean doctors do not always mention cause of death as HIV/AIDS on death certificates due to stigma, bias and underreporting [28, 29], this could possibly explain the sharp increase in endocrine disease mortality from 2004. Ischaemic heart disease has previously been found to be a less common cause of heart failure and a more limited problem in terms of burden in SSA [30, 31], explaining why it made the lowest contribution to CMD mortality in our results.

The increased risk of CMD is multifaceted and includes a combination of many factors such as urbanisation, HIV/AIDS treatment, and tuberculosis (TB). Pulmonary heart disease and CVD, made the largest contribution to CMD mortality. This result supports existing literature, as the percentage of premature deaths from CVDs is as high as 42 % in low-income countries [32]. Mortality attributable to CVD and pulmonary heart disease could be at least partly explained by infectious diseases namely; HIV/AIDS and tuberculosis. As Zimbabwe is experiencing an HIV/AIDS epidemic [33], the use of ART to treat HIV has led to an increase in metabolic dysfunction, including dyslipidemia and lipodystrophy [34], both risk factors for developing heart disease. Pulmonary hypertension, cardiomyopathy and pericardial disease, generally related to TB which is highly prevalent in Zimbabwe [33], are the most commonly reported cardiac manifestations in HIV/AIDS [34–36].

While this paper provides the first robust evidence of increasing CMD mortality, it does raise the question: what is causing the increase in CMD mortality in Zimbabwe?

Similar to many countries in SSA, significant social, cultural, and economic factors are contributing to the growing prevalence of obesity, hypertension and diabetes in Zimbabwe. Due to urbanization, families and individuals in SSA have shifted to more western, unhealthy diets consisting of high caloric intake and large amounts of refined carbohydrates and fats [37]. Causes of overweight and obesity have been linked to the consumption of high caloric food and sugar-sweetened beverages, of which sales of sugar-sweetened beverages are increasing worldwide, particularly in low- and middle-income countries as a result of heavy marketing [38, 39]. In Zimbabwe, the overall percentage of obesity in the population was 7 %, with 11.6 % of women and 2.4 % of men obese [11].

Obesity is a major risk factor for a number of CMDs, including hypertension, diabetes, and cardiovascular diseases [40, 41].

The health effects of this dietary change are compounded by the cultural expectations regarding food and body image, as fatness is associated with health and prosperity, and weight loss is generally undesirable because it raises suspicion that a person has a serious illness [42]. Furthermore, there are barriers to physical exercise that make weight loss difficult. Research has shown that physically inactivity is high in SSA [43] due to rapid urbanisation and socio-economic transitions [44, 45].

Another possible reason could be the absence of well-developed programmes for identification, comprehensive CMD risk assessment and management of high-risk individuals in Zimbabwe. Community based and high-risk prevention strategies and policies have been shown to reduce circulatory system mortality rates in developed countries, but also in some developing countries [46-49].

This study had limitations. Firstly, the utilization of national death registry data are limited by the under- or mis-reporting of specific cause of death on death certificates. Death certificates can be unreliable indicators of the causes of mortality, and mortality associated with cardiometabolic diseases are generally underestimated when only a single cause of death is coded, as in Zimbabwe [29, 50, 51].

Studies have reported that diabetes was listed as the underlying cause of death for only 7.7 % of diabetic men and 13.4 % of diabetic women [52, 53]. This could explain why the mortality rate for diabetes is lower than CVD and pulmonary heart disease. Behavioural, medical and socioeconomic factors have been shown to have a significant increase on mortality [54, 55], and the lack of these predictors in our mortality projection is a significant limitation. Therefore the mortality statistics likely underestimate the impact of cardiometabolic disease related deaths in Zimbabwe over the study period.

Secondly, in Zimbabwe 67 % of the population resides in rural areas, and it is likely a high proportion of all deaths occurred at home due to the inability to access healthcare services. Where only the mode of dying is reported on the death certificates (for example, “natural cause”) then a potentially wide range of different underlying causes are missed. The quality of the mortality data is determined largely by the medical knowledge, diligence and integrity of the certifying doctor, nurse or, in some rural areas, traditional chiefs or headman.

Studies seeking to identify areas to optimize for ICD-10 specificity should replicate the application in ICD-9 and analyze the results [51], as preliminary comparability ratios by cause of death have indicated an effect in the ranking of leading causes of death, as well as substantial discontinuities in cause-of death trends, such as diseases of the urinary system, resulting from implementing ICD–10 [56]. As the coding practices differed over the study period, this confers challenges in comparison and interpretation of disease codes which in turn underestimates the mortality of some of the diseases analyzed [57].

## Conclusion

This study has found that deaths due to CMD increased significantly over the period 1996-2007 in both males and females in Zimbabwe, and our projections suggest CMD mortality will substantially increase from 2015 to 2040. The most significant needs involve educating and effectively treating people with CMDs, with emphasis on co-morbid conditions such as HIV and TB, to substantially reduce CMD-related mortality. Zimbabwe’s healthcare system heavily focuses on tertiary care, with little attention to prevention. Further research into CMD morbidity and mortality in Zimbabwe is required to accurately address the causes and specific areas for health policy prevention strategies.

## Abbreviations

AIDS: Acquired Immune Deficiency Syndrome
CMD: Cardiometabolic disease
CVD: Cardiovascular disease
GAM: General additive model
ART: Antiretroviral therapy
HIV: Human Immunodeficiency Virus
ICD: International Classification of Disease version
MetS: Metabolic syndrome
MoHCC: Ministry of Health and Child Care
NCD: Non Communicable Disease
SSA: Sub-Saharan Africa
WHO: World Health Organization
ZimStat: Zimbabwe National Statistics Agency
